# Genetically Predicted PD1 and the Risk of Cardiovascular Diseases

**DOI:** 10.1111/jcmm.70678

**Published:** 2025-08-19

**Authors:** Wenfei Chen, Kangnan Wang, Jun Chen, Lei Chen

**Affiliations:** ^1^ Taizhou Central Hospital, the Affiliated Hospital of Taizhou College Taizhou Zhejiang China; ^2^ Zhejiang Chinese Medical University Hangzhou Zhejiang China; ^3^ Department of Cardiology Zhejiang Provincial People's Hospital, Affiliated People's Hospital, Hangzhou Medical College Zhejiang China

**Keywords:** cardio‐oncology, cardiovascular diseases, genetic evidence, immune checkpoint inhibitors, immunotherapy, PD1

## Abstract

Genetically speaking, ICI was mainly referred to as PD‐1. We utilized a two‐sample MR analysis model to assess the causative impact of PD1 on six kinds of CVDs, including coronary atherosclerosis, atrial fibrillation (AF), myocarditis, hypertrophic cardiomyopathy, dilated cardiomyopathy, and heart failure (HF). In this study, the IVW model as the primary MR approach revealed that genetically predicted PD1 has a causal impact on the increased risk of coronary atherosclerosis (OR, 1.062; 95% CI, 1.031–1.095, *p* = 6.81E‐05), as well as myocarditis (OR, 1.177; 95% CI, 1.010–1.371, *p* = 0.037). However, genetically predicted PD1 does not significantly increase the risk of AF (OR, 1.024; 95% CI, 0.969–1.082, *p* = 0.398), HF (OR, 1.025; 95% CI, 0.971–1.081, *p* = 0.371), dilated cardiomyopathy (OR, 0.924; 95% CI, 0.819–1.044, *p* = 0.207), and hypertrophic cardiomyopathy (OR, 1.047; 95% CI, 0.838–1.308, *p* = 0.685). Our study found that genetically predicted PD1 has a causal impact on the increased risk of coronary atherosclerosis and myocarditis.

**Abbreviations**
AF: atrial fibrillation
CI: confidence intervals
CVD: cardiovascular disease
GWAS: genome‐wide association studies (GWAS)
HF: heart failure
ICIs: immune checkpoint inhibitors
MR: Mendelian Randomization
SNP: single‐nucleotide polymorphism
TNFα: TUMOUR necrosis factor α

## 1 Introduction

Cardiovascular disease and cancer are the two primary reasons for morbidity and mortality worldwide. Cardio‐oncology, as an emerging subject, has indicated a cross‐connection between two disparate kinds of disease. These associations result from several factors: anti‐cancer treatment‐related cardiovascular injury, risk factors predisposing humans to cardiovascular disease and cancer and underlying cancer growth by cardiac dysfunction [1]. Nowadays, immune checkpoint inhibitors (ICIs) become a novel anti‐cancer strategy and have a promising effectiveness in many cancers. They resist tumour cells to achieve survival improvement by obstructing inhibitory molecules to elevate the *T*‐cell–mediated immune response. ICIs comprise programmed death 1 inhibitors (PD1), programmed death ligand 1 inhibitors (PDL1) and cytotoxic T‐lymphocyte–associated antigen 4 inhibitors (CTLA‐4) [2]. In recent years, numerous studies have reported that the adverse events of immune checkpoint therapies are associated with an increased risk of multiple cardiovascular diseases (CVDs), including myocarditis, atherosclerosis and arrhythmia [3]. However, observational studies are challenging to assess the causation of immune checkpoint inhibitors on CVDs due to possible confounding biases. Mendelian Randomization (MR) analysis helps to determine the causal relationship between risk factors and disease by using genetic variants as instrumental variables for risk factors. Due to the random assignment of the genetic variants at conception, MR analysis can minimise unmeasured confounders, which is a significant drawback of data from observational research. This study used MR analysis to assess the causal relationship between immune checkpoint inhibitors and several prevalent CVDs.

Genetically speaking, ICI was mainly referred to as PD‐1. We utilised a two‐sample MR analysis model to assess the causative impact of PD1 on six kinds of CVDs, including coronary atherosclerosis, atrial fibrillation (AF), myocarditis, hypertrophic cardiomyopathy, dilated cardiomyopathy and heart failure (HF). We extracted genetic instruments of PD‐1 targets from the INTERVAL study [4]. We included genetic variants steadily associated with PD‐1 according to the genome‐wide significance threshold (*p* < 5 × 10^−6^ and linkage disequilibrium clumping *r* ^2^ < 0.01). We screened the genetic instruments to guarantee the robustness of genetic variants related to PD‐1.

## 2 Patients and Methods

We obtained summary statistics on the risk of coronary atherosclerosis from GWAS summary data (ICD: ‘finn‐b‐I9_CORATHER’), AF dataset from GWAS summary data (ICD: ‘finn‐b‐I9_AF’), myocarditis dataset from GWAS summary data (ICD: ‘finn‐b‐I9_MYOCARD’), hypertrophic cardiomyopathy dataset from GWAS summary data (ICD: ‘finn‐b‐I9_HYPERTROCARDMYOP’), dilated cardiomyopathy dataset from GWAS summary data (ICD: ‘ebi‐a‐GCST90018834’) and HF dataset from GWAS summary data (ICD: ‘finn‐b‐I9_HEARTFAIL’). For the processing of palindromic SNPs, the main method is to discard those SNPs with palindromic structure. This is because palindromic sequences can cause confusion in genetic analysis, especially when trying to infer allele frequencies. According to the way the Harmonize software processes it, if a SNP has a palindromic structure and its allele frequency information no longer provides information about the chain, this will result in an inability to perform a valid genetic analysis. Therefore, we deleted the palindromic sequence before determining the IVs. We gauged the strength of the link between these IVs and exposure by computing their *F*‐statistics (*F*‐statistics = beta.exposure^2^/standard error.exposure^2^). SNPs presenting *F*‐statistics < 10 were discarded as larger *F*‐statistics suggest the genetic markers cause phenotypic variations. The formula for determining *R* ^2^ specifically is as follows: *R* ^2^ is equal to 2 × minor allele frequency (MAF) × (1‐MAF) × beta.exposure^2^.

### 2.1 Statistical Analysis

The random‐effects IVW approach was used to carry out the primary MR analysis. We applied MR–Egger regression and weighted median methods to assess the robustness of the results due to the presence of horizontal pleiotropy in the IVW estimates. We evaluated the heterogeneity by Cochran's *Q* statistics and IVW procedures. The MR Egger intercept test assessed the pleiotropy of each unique single‐nucleotide polymorphism (SNP). Statistical significance was defined as a two‐tailed *p* value of 0.05. We also performed a sensitivity analysis using the stricter threshold (*p* < 5 × 10^−8^), in this part, only one SNP (rs2330106) was associated with genetically predicted PD1; therefore, we used the wald ratio method to analyse the genetically predicted PD1 and the risk of cardiovascular diseases.

### 2.2 Ethics

Relevant institutional review boards had approved the study's ethical conduct before they were included in these GWAS meta‐analyses. This study only used the publicly available data from these investigations. Therefore, we did not necessarily require additional ethics approval. All data were summary‐level and de‐identified from the GWAS‐related database.

## 3 Results

The summary of genome‐wide association studies included in this study as shown in the Table 1. In this study, a total of 18 SNPs met the above requirements and included in the final analysis. The *F*‐statistics is among 19.903–87.955. The IVW model as the primary MR approach revealed that genetically predicted PD1 has a causal impact on the increased risk of coronary atherosclerosis (OR_ivw_, 1.062; 95% CI, 1.031–1.095, P_ivw_ = 6.81E‐05), as well as myocarditis (OR_ivw_, 1.177; 95% CI, 1.010–1.371, P_ivw_ = 0.037).

**TABLE 1:** Summary of genome‐wide association studies included in this study.

Phenotype	Cohort(s)	Sample size	Race	GWAS data source
PD‐1	European population	About 50,000	European	INTERVAL BioResource
Coronary atherosclerosis	European population	23,363 (ncase) 195,429 (ncontrol)	European	finn‐b‐I9_CORATHER
Atrial fibrillation	European population	22,068 (ncase) 196,724 (ncontrol)	European	finn‐b‐I9_AF
Myocarditis	European population	829 (ncase) 116,926 (ncontrol)	European	finn‐b‐I9_MYOCARD
Hypertrophic cardiomyopathy	European population	556 (ncase) 218,236 (ncontrol)	European	finn‐b‐I9_HYPERTROCARDMYOP
Heart failure	European population	13,087 (ncase) 195,091 (ncontrol)	European	finn‐b‐I9_HEARTFAIL
Dilated cardiomyopathy	European population	1444 (ncase) 355,381 (ncontrol)	European	ebi‐a‐GCST90018834

**FIGURE 1:**
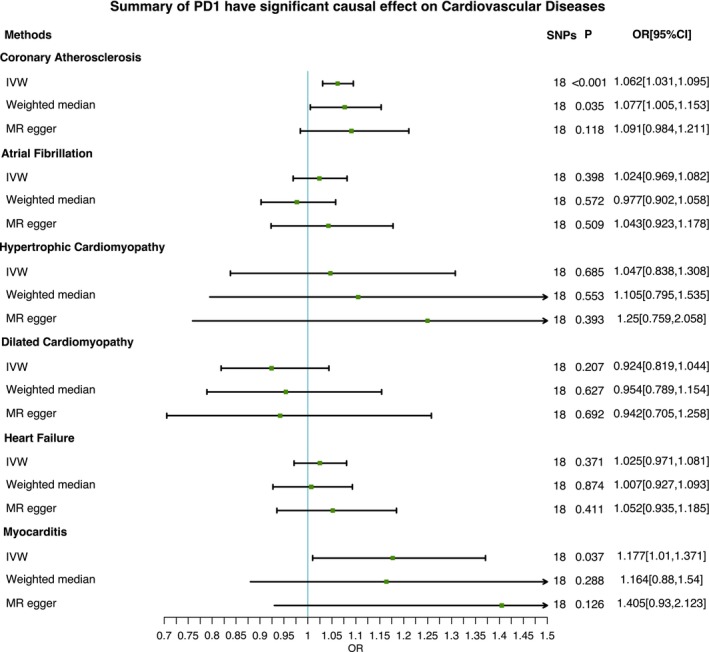
Forest plot for the cause–effect of PD1 on cardiovascular diseases.

However, genetically predicted PD1 does not significantly increase the risk of AF (OR_ivw_, 1.024; 95% CI, 0.969–1.082, P_ivw_ = 0.398), HF (OR_ivw_, 1.025; 95% CI, 0.971–1.081, P_ivw_ = 0.371), dilated cardiomyopathy (OR_ivw_, 0.924; 95% CI, 0.819–1.044, P_ivw_ = 0.207) and hypertrophic cardiomyopathy (OR_ivw_, 1.047; 95% CI, 0.838–1.308, P_ivw_ = 0.685). Figure 1 presents the detailed information. Cochran's Q statistics indicated no heterogeneity evidence in the study outcome. MR Egger's intercept test, an examination of the individual SNPs' pleiotropy, showed no impact of individual SNPs' pleiotropy on this study's results, the results as shown in Table S1. The schematic diagram results of genetically predicted PD1 on each CVD are shown in Figure S1–S6

In the sensitivity analysis of this study (if we set the stricter threshold (*p* < 5 × 10^−8^)), a total of one SNP (rs2330106) met the above requirements and was included in the final analysis. The Wald ratio model as the primary MR approach revealed that genetically predicted PD1 has a causal impact on the increased risk of coronary atherosclerosis (OR, 1.092; 95% CI, 0.974–1.225, *p* = 0.131), as well as myocarditis (OR, 1.181; 95% CI, 0.752–1.855, *p* = 0.470), dilated cardiomyopathy (OR, 1.136; 95% CI, 0.822–1.570, *p* = 0.439) and hypertrophic cardiomyopathy (OR, 1.391; 95% CI, 0.804–2.406, *p* = 0.239); however, there was no statistical difference. Genetically predicted PD1 was associated with the decreased risk of AF (OR, 0.993; 95% CI, 0.969–1.077, *p* = 0.874). As no corresponding SNP (rs2330106) could be found from the HF GWAS dataset (ICD: ‘finn‐b‐I9_HEARTFAIL’), we were unable to conduct the subsequent MR analysis.

## 4 Discussion

With the increasing clinical utilisation and significant beneficial prognosis of ICIs, it is important to pay attention to the toxicity or adverse drug impact on other body systems. Anti‐PD1 therapy disrupts cardiac immunity's homeostasis to injure myocardial function's integrity early. Cardiovascular‐related adverse events lead to potential issues in the prognosis of patients with ICIs.

Tumour necrosis factor α (TNFα) block is probably a usable approach to prevent the performance of ICI‐related cardiotoxicity [5]. Our study found that genetically predicted PD1 has a causal impact on the increased risk of coronary atherosclerosis and myocarditis. Even though the mechanism of ICI‐related cardiotoxicity is not explicit, some evidence recommends an effect for the robust proliferation and stimulation of *T* cell expression common, high‐frequency receptors against antigens shared by the tumour and affected tissue [6]. It has been discovered that *T* cells are derived from the same clones in both the tumour and the inflamed myocardium after immunotherapy [6]. *T*‐cell activation is also integral to atherogenesis. The infiltrating immune cells consist of CD4+ *T* cells, CD8+ *T* cells and CD68+ macrophages, indicating acute cellular rejection after cardiac transplantation and giant cell myocarditis [7]. Some additional reported mechanisms of ICI‐associated cardiovascular adverse actions cover auto‐antibodies elevation targeting self‐antigens (e.g., cardiac troponin, myosin, cardiac β1 adrenergic receptors) and *T* cell upregulation reacting with common antigens between cancer and normal cells (such as surface antigens expressed by cardiomyocytes or skeletal muscle) [7]. One recent meta‐analysis which included 14 eligible studies found that ICI leads to increased plaque inflammation and a significant increase in murine atherosclerotic plaque size. These changes may indicate that patients treated with ICIs were associated with an increased risk of myocardial infarction [8]. Furthermore, PD‐1 is important in regulating these immune processes within the cardiovascular system. These checkpoints regulate the activation and proliferation of immune cells, ensuring that inflammation does not become excessive and that self‐tolerance is maintained. Dysregulation of immune checkpoint pathways can result in excessively activated inflammatory responses, contributing to atherosclerosis and myocarditis. In atherosclerosis, ICI can exacerbate inflammation and promote plaque instability. However, in myocarditis, the excessive activation of immune cells can lead to myocardial injury and fibrosis [9].

However, our study did not find that genetically predicted PD1 has a causal impact on the increased risk of AF, HF, dilated cardiomyopathy and hypertrophic cardiomyopathy. Therefore, clinicians need to monitor the patients' chest pain symptoms and serum troponin levels during ICI therapy, especially the patients with a history of coronary atherosclerosis or myocarditis.

This study had several limitations. Firstly, genetic proxies for PD‐1 expression model a predisposition, not drug exposure of ICI per se. Therefore, when further explaining the causal relationship between ICI and cardiovascular diseases, it is necessary to take into account that our results are only a subset of the above‐mentioned relationship. Secondly, no colocalisation analysis was performed to ensure that the signal for PD‐1 expression overlaps genetically with CVD risk loci. Thirdly, the borderline clinical significance (OR is 1.062 for coronary atherosclerosis) was observed in the causal relationship at the genetic PD‐1 with coronary atherosclerosis, these may need cautious interpretation and need to be further confirmed in future researches. The generalisability of our findings to specific ethnic groups needs to be confirmed, as our analysis primarily focused on European populations.

## Author Contributions

**Wenfei Chen:** investigation (equal), writing – original draft (equal). **Kangnan Wang:** writing – review and editing (equal). **Jun Chen:** investigation (equal), methodology (equal), validation (equal). **Lei Chen:** conceptualization (equal), writing – review and editing (equal).

## Ethics Statement

Our research was conducted entirely on publicly available, anonymised data. Therefore, individual patient consent was not required. All methods followed relevant guidelines to protect the patient's privacy.

## Consent

The authors have nothing to report.

## Conflicts of Interest

The authors declare no conflicts of interest.

## Supporting information


**Figure S1.** (A) Scatter plot to visualise the causal effect of PD‐1 on dilated cardiomyopathy. The slope of the straight line indicates the magnitude of the causal association; (B) IVW analysis of the causal association of PD‐1 with dilated cardiomyopathy. The black dots and bars indicated the causal estimate and 95% CI using each SNP. The red dot and bar indicated the overall estimate and 95% CI meta‐analysed by MR–Egger and inverse‐variance weighted method; (C) MR leave‐one‐out sensitivity analysis for PD‐1 on dilated cardiomyopathy. Circles indicate MR estimates for PD‐1 on dilated cardiomyopathy using inverse‐variance weighted if each SNP was omitted in turn. (D) *funnel* plot to visualise the causal effect of PD‐1 on dilated cardiomyopathy.
**Figure S2**. (A) Scatter plot to visualise the causal effect of PD‐1 on AF. The slope of the straight line indicates the magnitude of the causal association; (B) IVW analysis of the causal association of PD‐1 with AF. The black dots and bars indicated the causal estimate and 95% CI using each SNP. The red dot and bar indicated the overall estimate and 95% CI meta‐analysed by MR–Egger and inverse‐variance weighted method; (C) MR leave‐one‐out sensitivity analysis for PD‐1 on AF. Circles indicate MR estimates for PD‐1 on AF using inverse‐variance weighted method if each SNP was omitted in turn. (D) *funnel* plot to visualise the causal effect of PD‐1 on AF.
**Figure S3**. (A) Scatter plot to visualise the causal effect of PD‐1 on coronary atherosclerosis. The slope of the straight line indicates the magnitude of the causal association; (B) IVW analysis of the causal association of PD‐1 with coronary atherosclerosis. The black dots and bars indicated the causal estimate and 95% CI using each SNP. The red dot and bar indicated the overall estimate and 95% CI meta‐analysed by MR–Egger and inverse‐variance weighted method; (C) MR leave‐one‐out sensitivity analysis for PD‐1 on coronary atherosclerosis. Circles indicate MR estimates for PD‐1 on coronary atherosclerosis using inverse‐variance weighted method if each SNP was omitted in turn. (D) *funnel* plot to visualise the causal effect of PD‐1 on coronary atherosclerosis.
**Figure S4**. (A) Scatter plot to visualise the causal effect of PD‐1 on heart failure. The slope of the straight line indicates the magnitude of the causal association; (B) IVW analysis of the causal association of PD‐1 with heart failure. The black dots and bars indicated the causal estimate and 95% CI using each SNP. The red dot and bar indicated the overall estimate and 95% CI meta‐analysed by MR–Egger and inverse‐variance weighted method; (C) MR leave‐one‐out sensitivity analysis for PD‐1 on heart failure. Circles indicate MR estimates for PD‐1 on heart failure using inverse‐variance weighted method if each SNP was omitted in turn. (D) *funnel* plot to visualise the causal effect of PD‐1 on heart failure.
**Figure S5**. (A) Scatter plot to visualise the causal effect of PD‐1 on hypertrophic cardiomyopathy. The slope of the straight line indicates the magnitude of the causal association; (B) IVW analysis of the causal association of PD‐1 with hypertrophic cardiomyopathy. The black dots and bars indicated the causal estimate and 95% CI using each SNP. The red dot and bar indicated the overall estimate and 95% CI meta‐analysed by MR–Egger and inverse‐variance weighted method; (C) MR leave‐one‐out sensitivity analysis for PD‐1 on hypertrophic cardiomyopathy. Circles indicate MR estimates for PD‐1 on hypertrophic cardiomyopathy using inverse‐variance weighted method if each SNP was omitted in turn. (D) *funnel* plot to visualise the causal effect of PD‐1 on hypertrophic cardiomyopathy.
**Figure S6**. (A) Scatter plot to visualise the causal effect of PD‐1 on myocarditis. The slope of the straight line indicates the magnitude of the causal association; (B) IVW analysis of the causal association of PD‐1 with myocarditis. The black dots and bars indicated the causal estimate and 95% CI using each SNP. The red dot and bar indicated the overall estimate and 95% CI meta‐analysed by MR–Egger and inverse‐variance weighted method; (C) MR leave‐one‐out sensitivity analysis for PD‐1 on myocarditis. Circles indicate MR estimates for PD‐1 on myocarditis using inverse‐variance weighted method if each SNP was omitted in turn. (D) *funnel* plot to visualise the causal effect of PD‐1 on myocarditis.

**Table S1.** Mendelian randomization estimates for PD‐1 on cardiovascular diseases.

## Data Availability

The datasets generated and analyzed during the current study are available from the GWAS database.
