# Supplementary material for: Genetically Predicted PD1 and the Risk of Cardiovascular Diseases
Source: J Cell Mol Med. 2025 Aug 19;29(16):e70678. doi: 10.1111/jcmm.70678 (PMC12364613; doi:10.1111/jcmm.70678)

**FIGURE LEGENDS**

Supplementary Figure 1 A. Scatter plot to visualize causal effect of PD-1 on dilated cardiomyopathy. The slope of the straight line indicates the magnitude of the causal association; Figure B. IVW analysis of the causal association of PD-1 with dilated cardiomyopathy. The black dots and bars indicated the causal estimate and 95% CI using each SNP. The red dot and bar indicated the overall estimate and 95% CI meta-analyzed by MR-Egger and inverse variance weighted method; Figure C. MR leave-one-out sensitivity analysis for PD-1 on dilated cardiomyopathy. Circles indicate MR estimates for PD-1 on dilated cardiomyopathy using inverse-variance weighted if each SNP was omitted in turn. Figure D. Funnel plot to visualize causal effect of PD-1 on dilated cardiomyopathy.


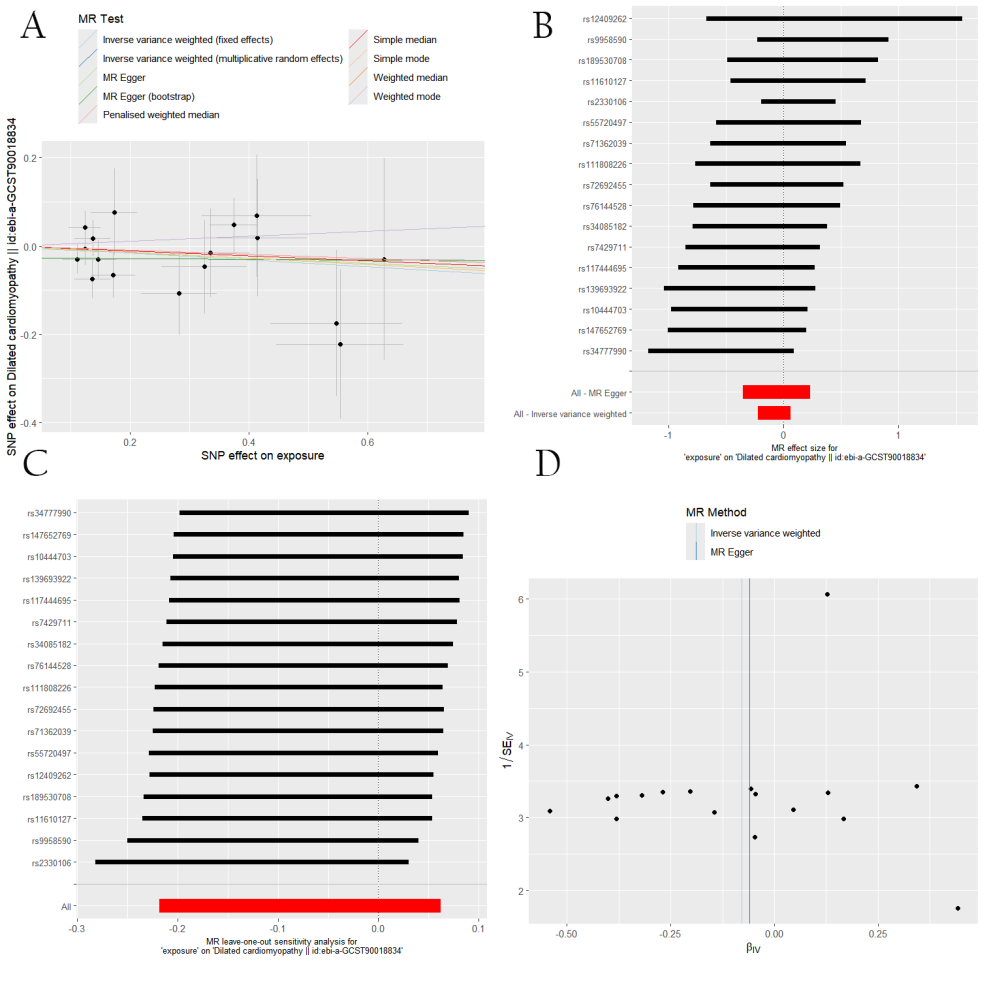


Supplementary Figure 2 A. Scatter plot to visualize causal effect of PD-1 on AF. The slope of the straight line indicates the magnitude of the causal association; Figure B. IVW analysis of the causal association of PD-1 with AF. The black dots and bars indicated the causal estimate and 95% CI using each SNP. The red dot and bar indicated the overall estimate and 95% CI meta-analyzed by MR-Egger and inverse variance weighted method; Figure C. MR leave-one-out sensitivity analysis for PD-1 on AF. Circles indicate MR estimates for PD-1 on AF using inverse-variance weighted method if each SNP was omitted in turn. Figure D. Funnel plot to visualize causal effect of PD-1 on AF.


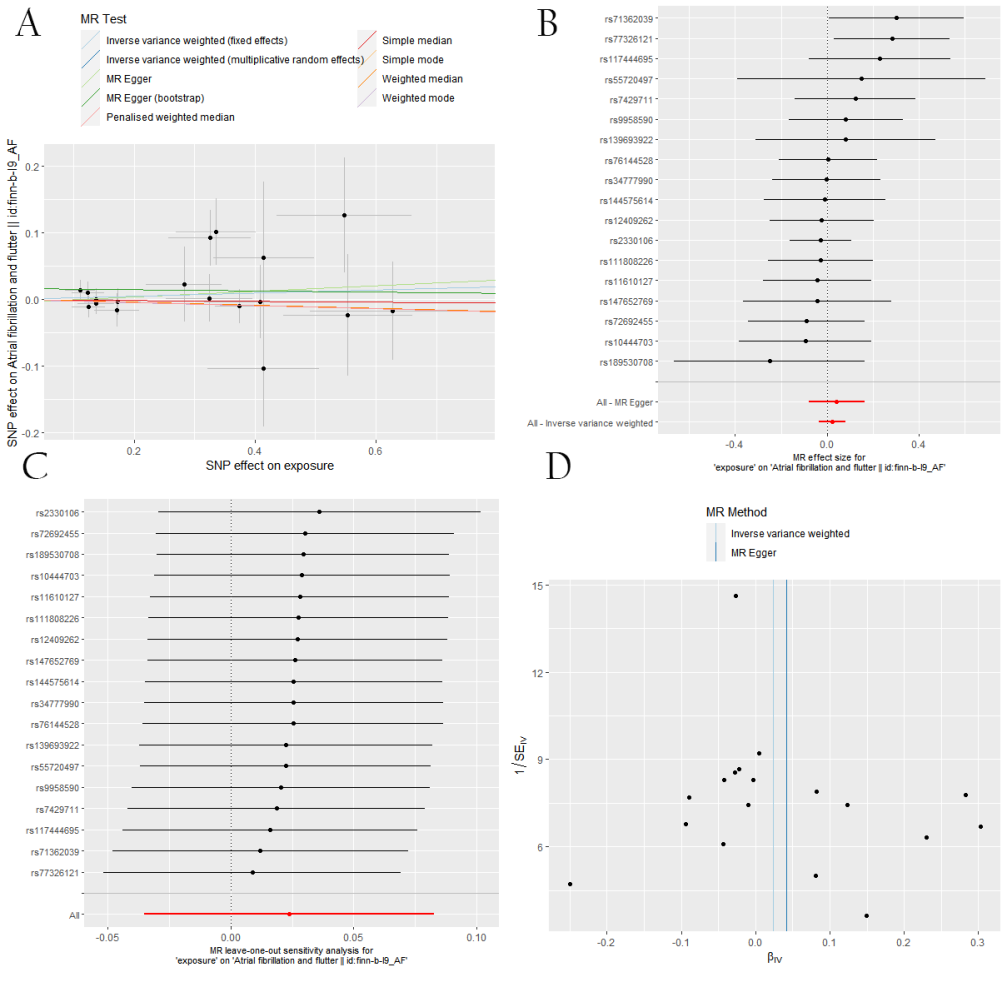


Supplementary Figure 3 A. Scatter plot to visualize causal effect of PD-1 on coronary atherosclerosis. The slope of the straight line indicates the magnitude of the causal association; Figure B. IVW analysis of the causal association of PD-1 with coronary atherosclerosis. The black dots and bars indicated the causal estimate and 95% CI using each SNP. The red dot and bar indicated the overall estimate and 95% CI meta-analyzed by MR-Egger and inverse variance weighted method; Figure C. MR leave-one-out sensitivity analysis for PD-1 on coronary atherosclerosis. Circles indicate MR estimates for PD-1 on coronary atherosclerosis using inverse-variance weighted method if each SNP was omitted in turn. Figure D. Funnel plot to visualize causal effect of PD-1 on coronary atherosclerosis.


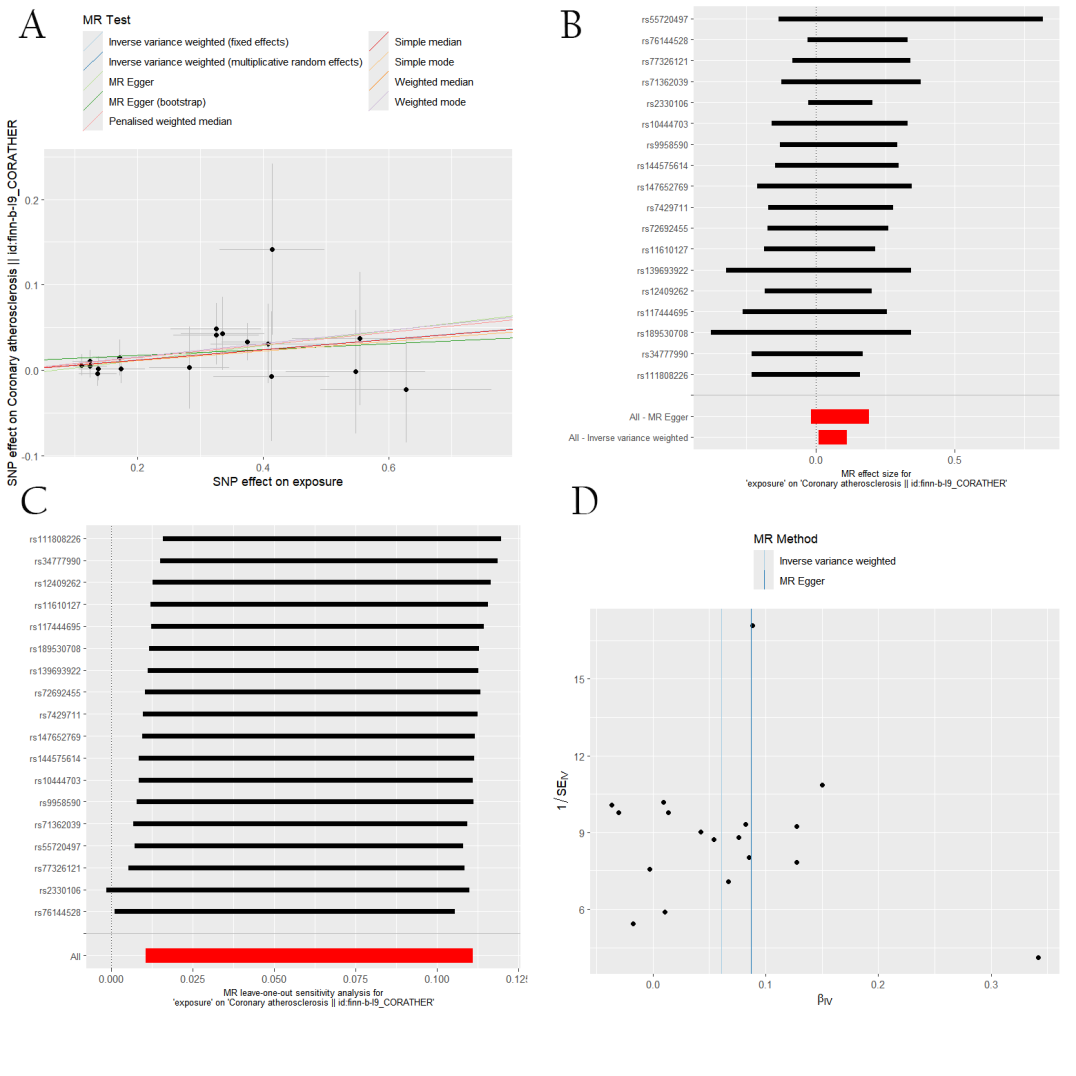


Supplementary Figure 4 A. Scatter plot to visualize causal effect of PD-1 on heart failure. The slope of the straight line indicates the magnitude of the causal association; Figure B. IVW analysis of the causal association of PD-1 with heart failure. The black dots and bars indicated the causal estimate and 95% CI using each SNP. The red dot and bar indicated the overall estimate and 95% CI meta-analyzed by MR-Egger and inverse variance weighted method; Figure C. MR leave-one-out sensitivity analysis for PD-1 on heart failure. Circles indicate MR estimates for PD-1 on heart failure using inverse-variance weighted method if each SNP was omitted in turn. Figure D. Funnel plot to visualize causal effect of PD-1 on heart failure.


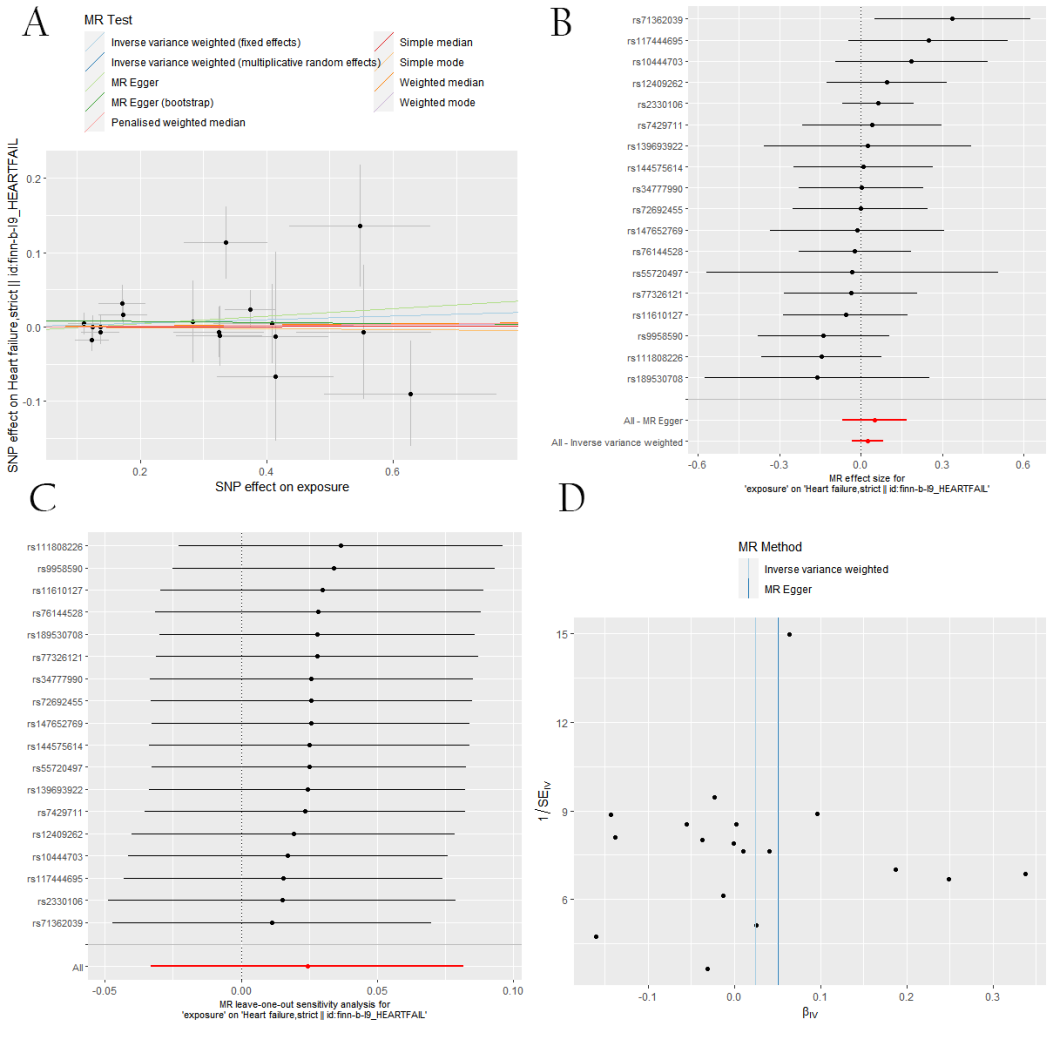


Supplementary Figure 5 A. Scatter plot to visualize causal effect of PD-1 on hypertrophic cardiomyopathy. The slope of the straight line indicates the magnitude of the causal association; Figure B. IVW analysis of the causal association of PD-1 with hypertrophic cardiomyopathy. The black dots and bars indicated the causal estimate and 95% CI using each SNP. The red dot and bar indicated the overall estimate and 95% CI meta-analyzed by MR-Egger and inverse variance weighted method; Figure C. MR leave-one-out sensitivity analysis for PD-1 on hypertrophic cardiomyopathy. Circles indicate MR estimates for PD-1 on hypertrophic cardiomyopathy using inverse-variance weighted method if each SNP was omitted in turn. Figure D. Funnel plot to visualize causal effect of PD-1 on hypertrophic cardiomyopathy.


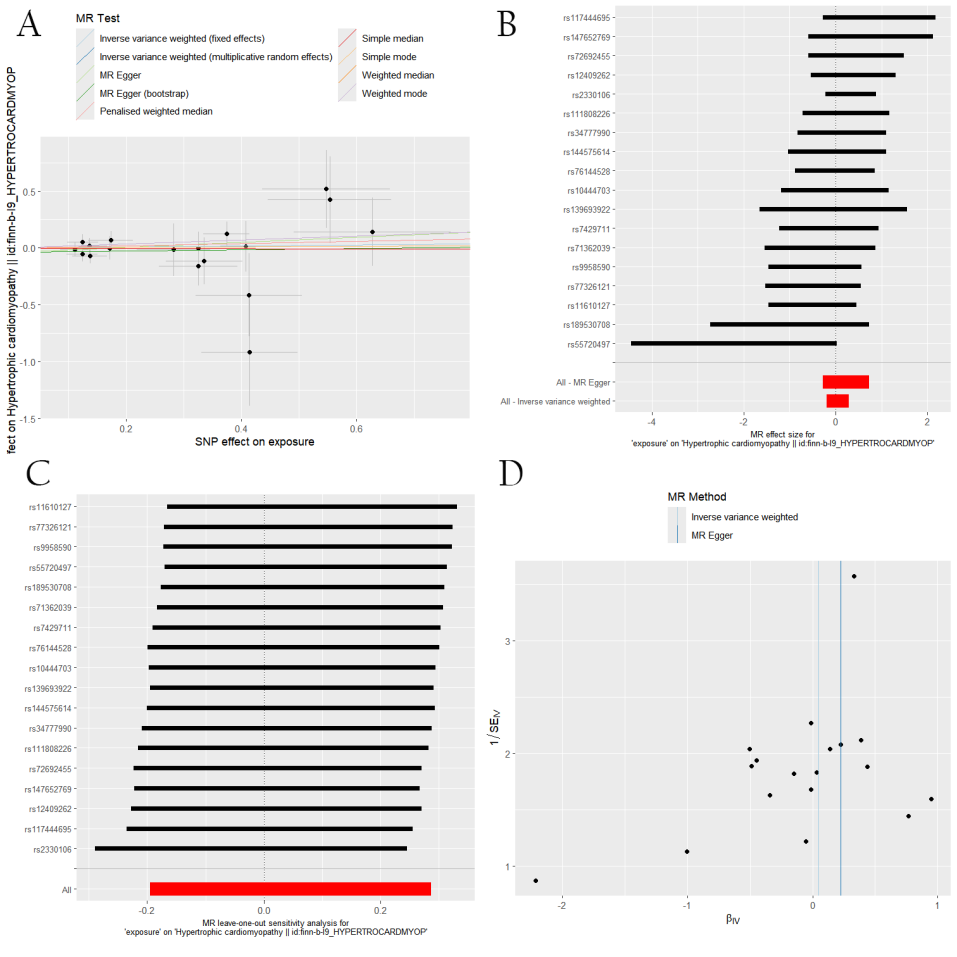


Supplementary Figure 6 A. Scatter plot to visualize causal effect of PD-1 on myocarditis. The slope of the straight line indicates the magnitude of the causal association; Figure B. IVW analysis of the causal association of PD-1 with myocarditis. The black dots and bars indicated the causal estimate and 95% CI using each SNP. The red dot and bar indicated the overall estimate and 95% CI meta-analyzed by MR-Egger and inverse variance weighted method; Figure C. MR leave-one-out sensitivity analysis for PD-1 on myocarditis. Circles indicate MR estimates for PD-1 on myocarditis using inverse-variance weighted method if each SNP was omitted in turn. Figure D. Funnel plot to visualize causal effect of PD-1 on myocarditis.


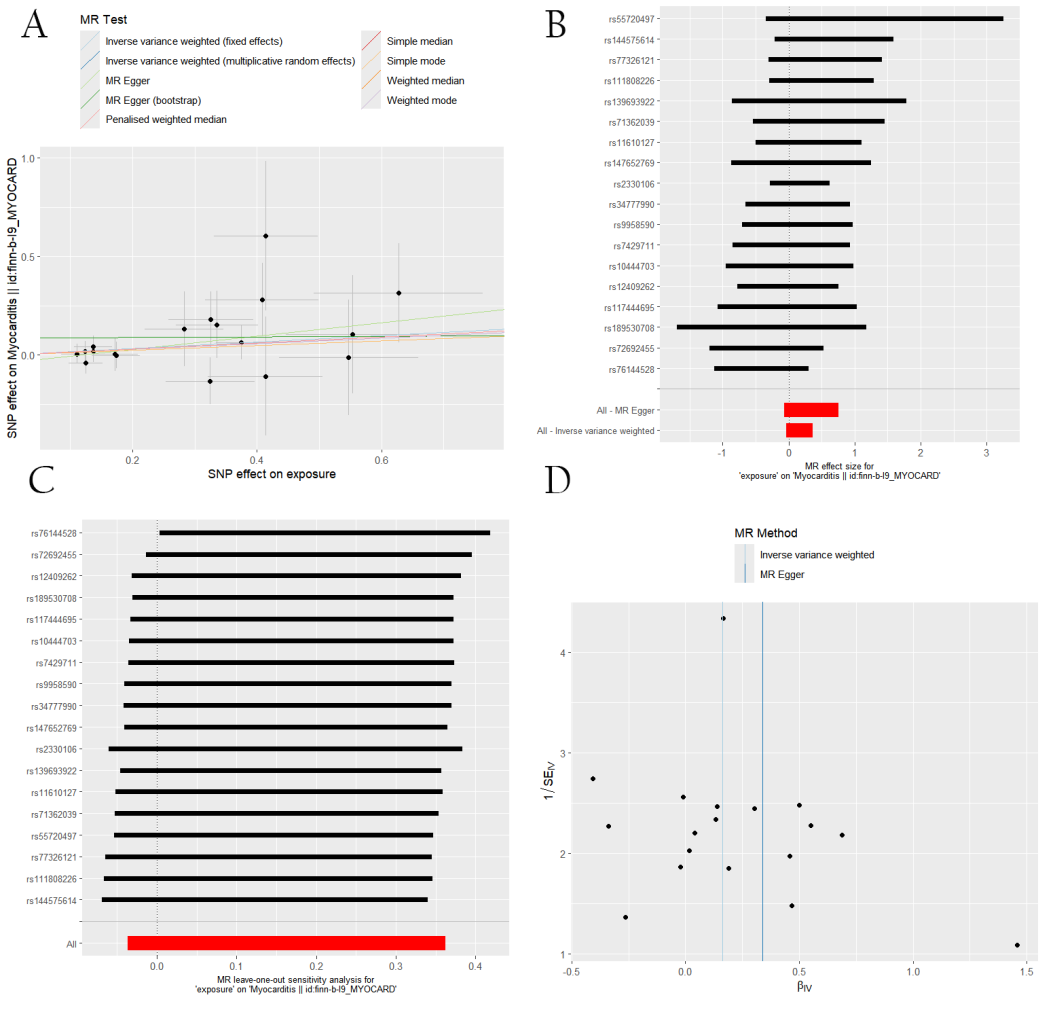

Supplement: Supplementary file 1 — Figure S1. (A) Scatter plot to visualise the causal effect of PD‐1 on dilated cardiomyopathy. The slope of the straight line indicates the magnitude of the causal association; (B) IVW analysis of the causal association of PD‐1 with dilated cardiomyopathy. The black dots and bars indicated the causal estimate and 95% CI using each SNP. The red dot and bar indicated the overall estimate and 95% CI meta‐analysed by MR–Egger and inverse‐variance weighted method; (C) MR leave‐one‐out sensitivity analysis for PD‐1 on dilated cardiomyopathy. Circles indicate MR estimates for PD‐1 on dilated cardiomyopathy using inverse‐variance weighted if each SNP was omitted in turn. (D) funnel plot to visualise the causal effect of PD‐1 on dilated cardiomyopathy. Figure S2. (A) Scatter plot to visualise the causal effect of PD‐1 on AF. The slope of the straight line indicates the magnitude of the causal association; (B) IVW analysis of the causal association of PD‐1 with AF. The black dots and bars indicated the causal estimate and 95% CI using each SNP. The red dot and bar indicated the overall estimate and 95% CI meta‐analysed by MR–Egger and inverse‐variance weighted method; (C) MR leave‐one‐out sensitivity analysis for PD‐1 on AF. Circles indicate MR estimates for PD‐1 on AF using inverse‐variance weighted method if each SNP was omitted in turn. (D) funnel plot to visualise the causal effect of PD‐1 on AF. Figure S3. (A) Scatter plot to visualise the causal effect of PD‐1 on coronary atherosclerosis. The slope of the straight line indicates the magnitude of the causal association; (B) IVW analysis of the causal association of PD‐1 with coronary atherosclerosis. The black dots and bars indicated the causal estimate and 95% CI using each SNP. The red dot and bar indicated the overall estimate and 95% CI meta‐analysed by MR–Egger and inverse‐variance weighted method; (C) MR leave‐one‐out sensitivity analysis for PD‐1 on coronary atherosclerosis. Circles indicate MR estimates for PD‐1 o [file JCMM-29-e70678-s001.docx]
