# Supplementary material for: Genetically Predicted PD1 and the Risk of Cardiovascular Diseases
Source: J Cell Mol Med. 2025 Aug 19;29(16):e70678. doi: 10.1111/jcmm.70678 (PMC12364613; doi:10.1111/jcmm.70678)
Supplement: Supplementary file 2 — Table S1. Mendelian randomization estimates for PD‐1 on cardiovascular diseases. [file JCMM-29-e70678-s002.docx]

**Supplementary Table 1 Mendelian randomization estimates for PD-1 on** **cardiovascular diseases.**

| Phenotype | N SNPs | Methods | OR 95% CI | P value | Cochran’s Q | P value | MR intercept | P value |
| --- | --- | --- | --- | --- | --- | --- | --- | --- |
| coronary atherosclerosis | 18 | IVW | 1.063 (1.031-1.095) | 6.813E-05 | 6.043 | 0.993 | -0.006 | 0.57 |
|  |  | Weighted median | 1.077 (1.005-1.153) | 0.035 | NA | NA |  |  |
|  |  | MR Egger | 1.091 (0.984-1.211) | 0.118 | 5.714 | 0.991 |  |  |
| atrial fibrillation | 18 | IVW | 1.024 (0.969-1.082) | 0.398 | 14.894 | 0.603 | -0.004 | 0.743 |
|  |  | Weighted median | 0.977 (0.902-1.058) | 0.572 | NA | NA |  |  |
|  |  | MR Egger | 1.043 (0.923-1.178) | 0.509 | 14.783 | 0.541 |  |  |
| myocarditis | 18 | IVW | 1.177（1.010-1.371） | 0.037 | 10.019 | 0.903 | -0.040 | 0.350 |
|  |  | Weighted median | 1.163（0.880-1.540） | 0.288 | NA | NA |  |  |
|  |  | MR Egger | 1.405（0.930-2.123） | 0.126 | 9.091 | 0.910 |  |  |
| hypertrophic cardiomyopathy | 18 | IVW | 1.047（0.838-1.308） | 0.685 | 14.527 | 0.629 | -0.040 | 0.438 |
|  |  | Weighted median | 1.105（0.795-1.535） | 0.553 | NA | NA |  |  |
|  |  | MR Egger | 1.250（0.759-2.058） | 0.393 | 13.895 | 0.607 |  |  |
| Heart failure | 18 | IVW | 1.025（0.971-1.081） | 0.370 | 14.699 | 0.617 | -0.006 | 0.621 |
|  |  | Weighted median | 1.007（0.927-1.093） | 0.874 | NA | NA |  |  |
|  |  | MR Egger | 1.052（0.935-1.185） | 0.411 | 14.445 | 0.565 |  |  |
| dilated cardiomyopathy | 17 | IVW | 0.925（0.819-1.044） | 0.207 | 11.932 | 0.686 | -0.004 | 0.888 |
|  |  | Weighted median | 0.954（0.789-1.154） | 0.627 | NA | NA |  |  |
|  |  | MR Egger | 0.942（0.705-1.258） | 0.692 | 11.911 | 0.749 |  |  |
